# Common and uncommon features of focal splenic lesions on contrast-enhanced ultrasound: a pictorial review

**DOI:** 10.1590/0100-3984.2015.0209

**Published:** 2017

**Authors:** Julia D. Zavariz, Eleni Konstantatou, Annamaria Deganello, Diana Bosanac, Dean Y. Huang, Maria E. Sellars, Paul S. Sidhu

**Affiliations:** 1 MD, Department of Radiology, Hospital das Clínicas da Faculdade de Medicina da Universidade de São Paulo (HC-FMUSP), São Paulo, SP, Brazil.; 2 MD, Department of Radiology, King's College Hospital, Denmark Hill, London, United Kingdom.; 3 FRCR, Department of Radiology, King's College Hospital, Denmark Hill, London, United Kingdom.

**Keywords:** Ultrasonography/methods, Microbubbles, Spleen/diagnostic imaging, Wounds and injuries, Neoplasms, Ultrassonografia/métodos, Microbolhas, Baço/diagnóstico por imagem, Ferimentos e lesões, Neoplasias

## Abstract

The characterization of focal splenic lesions by ultrasound can be quite challenging. The recent introduction of contrast-enhanced ultrasound (CEUS) has come to play a valuable role in the field of imaging splenic pathologies, offering the possibility of an ionizing radiation-free investigation. Because CEUS has been incorporated into everyday clinical practice, malignant diseases such as focal lymphomatous infiltration, metastatic deposits, benign cysts, traumatic fractures, and hemangiomas can now be accurately depicted and characterized without the need for further imaging. More specifically, splenic traumatic fractures do not require additional imaging by computed tomography (with ionizing radiation exposure) for follow-up, because splenic fractures and their complications are safely imaged with CEUS. In the new era of CEUS, more patients benefit from radiation-free investigation of splenic pathologies with high diagnostic accuracy.

## Abstract (pt)

A caracterização de lesões focais esplênicas pela ultrassonografia pode ser bastante desafiadora. A introdução da ultrassonografia com contraste por microbolhas vem ganhando papel importante no campo da avaliação por imagem das doenças esplênicas, oferecendo um método livre de radiação ionizante. Após a implementação da ultrassonografia contrastada na prática médica, doenças malignas como linfomas e metástases, bem como benignas, como cistos, lesões traumáticas e hemangiomas, podem ser observadas e caracterizadas de maneira acurada, sem a necessidade de prosseguir a investigação com outros métodos de imagem. Mais especificamente, lesões traumáticas esplênicas podem ser acompanhadas por meio da ultrassonografia contrastada, evitando a radiação ionizante da tomografia computadorizada, uma vez que as fraturas esplênicas e suas potenciais complicações são seguramente demonstradas por esse método ultrassonográfico. Na nova era do uso dos contrastes para ultrassonografia, mais pacientes serão beneficiados por investigações livres de radiação para avaliação de afecções do baço, com alta acurácia diagnóstica.

## INTRODUCTION

The search for efficient and less expensive imaging techniques is an ongoing challenge worldwide, ultrasound being ideally positioned to provide comprehensive imaging in a cost-effective manner. Ultrasound is a valuable tool in the study of the spleen, with the ability to determine and monitor alterations in the size, presence, and character of focal lesions, as well as diffuse alterations in the echo pattern. In the last 20 years, contrast-enhanced ultrasound (CEUS) has seen advances in several aspects ^(1)^. Its use for the examination of the spleen has been established in guidelines ^(1,2)^ and documented in specific studies ^(3-5)^. The combination of microbubble contrast and ultrasound minimizes three challenges faced in imaging: the deleterious effects of the ionizing radiation employed in computed tomography (CT); the possibility of nephrotoxicity caused by the iodinated contrast agents used in CT and magnetic resonance imaging (MRI); and the relatively high costs of CT and MRI. Here, we present a pictorial review of the CEUS presentations of a variety of splenic lesions, in comparison with those visible on B-mode ultrasound, CT, and MRI.

## CEUS

The contrast agents used in ultrasound are composed of small (3-5 µm) microbubbles, with low solubility in blood, covered by a membrane-typically a lipid, although proteins and polymers are also used ^(3)^. These microbubbles are small enough to pass through the lung capillaries but much larger than the molecules and particles used in CT and MRI contrast agents. Therefore, these microbubbles do not cross the endothelium and cell membrane, remaining exclusively in the intravascular space. The microbubble ultrasound contrast agent in current use is not nephrotoxic, and its excretion is essentially pulmonary, the inert gas (sulfur hexafluoride) being excreted via the respiratory tract and the phospholipid shell being metabolized in the liver.

Severe adverse events related to the use of microbubble contrast agents, anaphylactic shock being the worst possible event, are rare, with an estimated prevalence of 0.0086%, considerably lower than that reported for the use of iodinated contrast agents and comparable to those reported for most antibiotics and analgesics ^(3)^. In a large review of the use of microbubble contrast in over 23,000 abdominal studies, there were no deaths and only two anaphylactic reactions occurred, the incidence of serious events being less than 1 event/10,000 examinations ^(6)^. The authors reported that the most common adverse effects were cough and back pain. Other possible effects are headache, nausea, and ventricular extrasystole, the last possibly being relevant only in cardiac examinations.

## ULTRASOUND TECHNIQUE

Only after the spleen has been examined with B-mode and Doppler ultrasound should a CEUS examination be performed. The operator must choose the best patient position to allow visualization during respiration, taking both patient and operator comfort into account. To improve visualization of the deep pole and subphrenic areas of the spleen, for example, the patient can be placed in the lateral decubitus position or imaged during inspiration (shallow, measured breaths are required for a lesion to be visualized constantly throughout the examination). For better image definition, we use the highest frequency possible, tailored to the depth of the target area. Although it is normal practice to use a 3.5-5.0 MHz transducer, resolution is improved with a 7.5 MHz transducer, albeit at the expense of the detection of less obvious microbubbles ^(3)^. At our institution, a Siemens Acuson S2000 ultrasound system (Siemens Medical Solutions; Mountain View, CA, USA) is used with the Lumason/SonoVue microbubble contrast agent (Bracco; Milan, Italy). The microbubble contrast agent is injected intravenously via a cannula inserted into the left antecubital fossa, at a dose of 1.2-2.4 mL; the dose recommended by the manufacturer is 4.8 mL, which is now rarely needed. With the improvements in the quality of the ultrasound systems, transducers, and image processing, 1.2 mL of microbubble contrast is sufficient for examination of the spleen, particularly in smaller patients. If necessary, the procedure can safely be repeated after a 5-min interval.

In all of the cases illustrated here, a low-mechanical-index technique-cadence contrast pulse sequencing (Cadence CPS; Siemens Medical Solutions)-was used, the mechanical index being set at or below 0.10, with split-screen imaging, which allows the targeting of focal lesions. The mechanical index is a measure of the power of the ultrasound beam, and a low mechanical index results in minimal microbubble destruction, thus allowing enhanced imaging for a prolonged period ^(3)^. During CEUS, a digital video clip is recorded and still images are acquired as necessary.

## CEUS OF THE NORMAL SPLEEN

**Figure 1:**
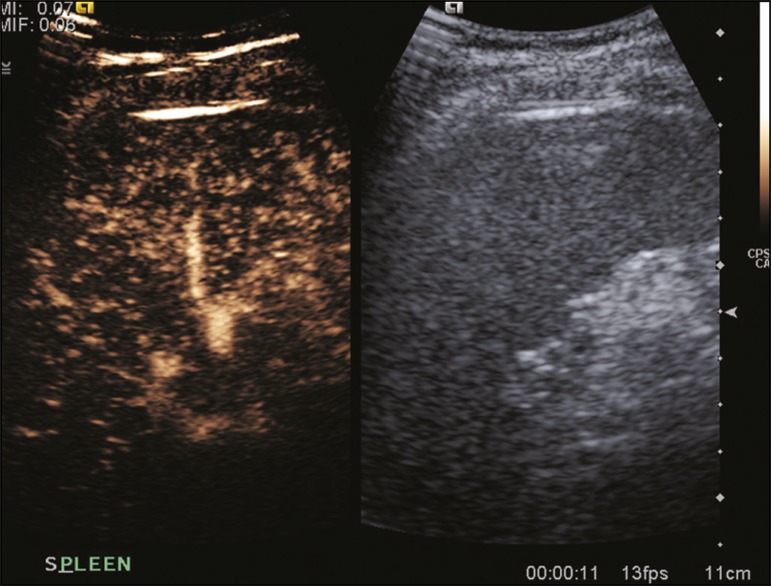
Zebra sign. A split-screen view. Striations are seen in a normally perfused spleen in the early arterial phase, the CT analog of the zebra sign.

The spleen is a solid organ, the superficial location of which makes it well suited for CEUS examination. On CEUS, the perfusion dynamics of the spleen differ from those of the liver, in which there is a dual blood supply, and mirrors the perfusion characteristics seen on a contrast-enhanced CT scan, although with better definition of the vascular components. In the early phase, microbubble contrast is seen within the splenic artery and its branches, usually within the first 20 s after microbubble contrast injection. Subsequently, the opacification becomes inhomogeneous, producing the so-called "zebra" pattern, which mimics what is seen in the arterial phase of CT (Figure 1). The splenic enhancement becomes homogeneous after approximately 60 s, and the overall enhancement lasts for 5-7 min.

## ABNORMALITIES OF THE SPLEEN ON CEUS

### Splenic cyst

**Figure 2:**
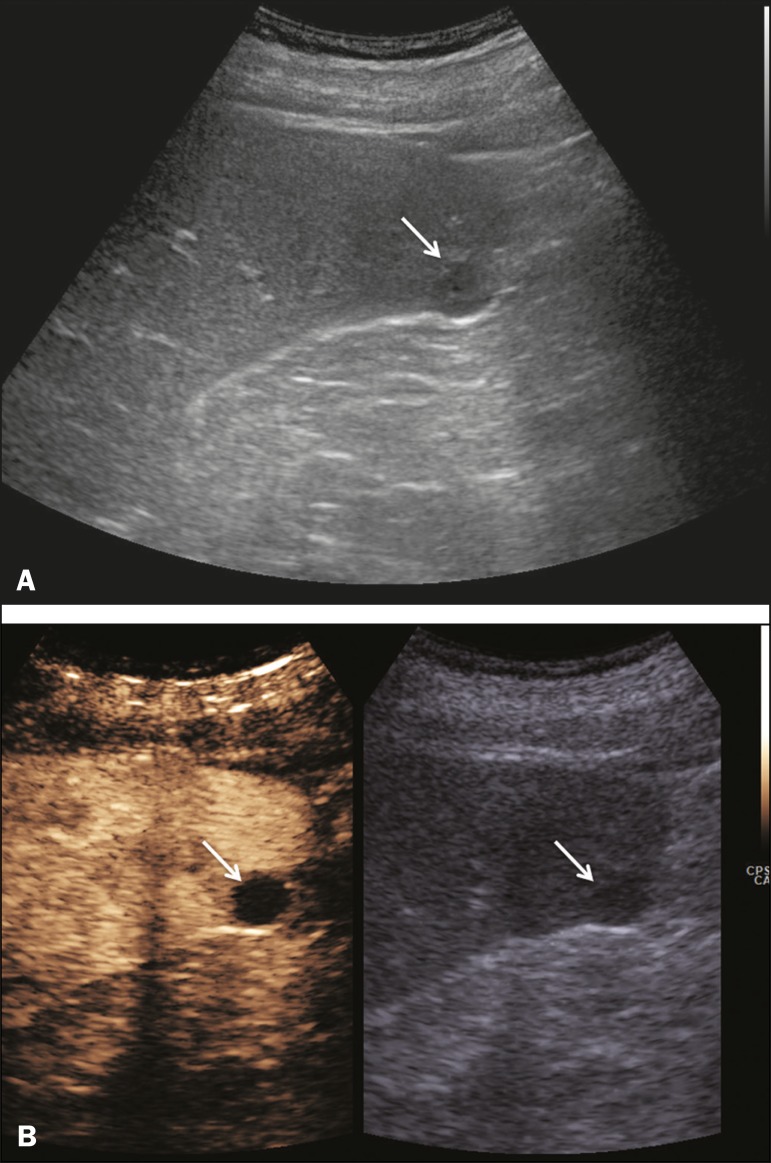
Simple cyst. **A:** B-mode ultrasound showing a well-circumscribed cystic structure (arrow) with a degree of a posterior acoustic enhancement. **B:** A split-screen view. The CEUS emphasized the features of the simple cyst (arrow), showing a well-circumscribed, thin-walled mass with minimal posterior acoustic enhancement.

Splenic cysts (Figure 2) can be primary (with cellular lining) or secondary. Most common primary cysts are simple, congenital, unilocular cysts, occurring most commonly in children and adolescents, predominantly in females. Less commonly, primary cysts are parasitic, hydatid disease being the leading cause of such cysts in endemic regions ^(7)^. Secondary cysts have no cellular lining and typically occur after trauma. Cysts can have internal debris, the echogenicity of which makes them difficult to evaluate on B-mode ultrasound, although primary congenital cysts are usually well defined, with posterior acoustic enhancement. Following microbubble contrast administration, the definition of the cyst is enhanced, no internal vascularity is observed, and any internal debris is not enhanced.

### Splenic abscess

**Figure 3:**
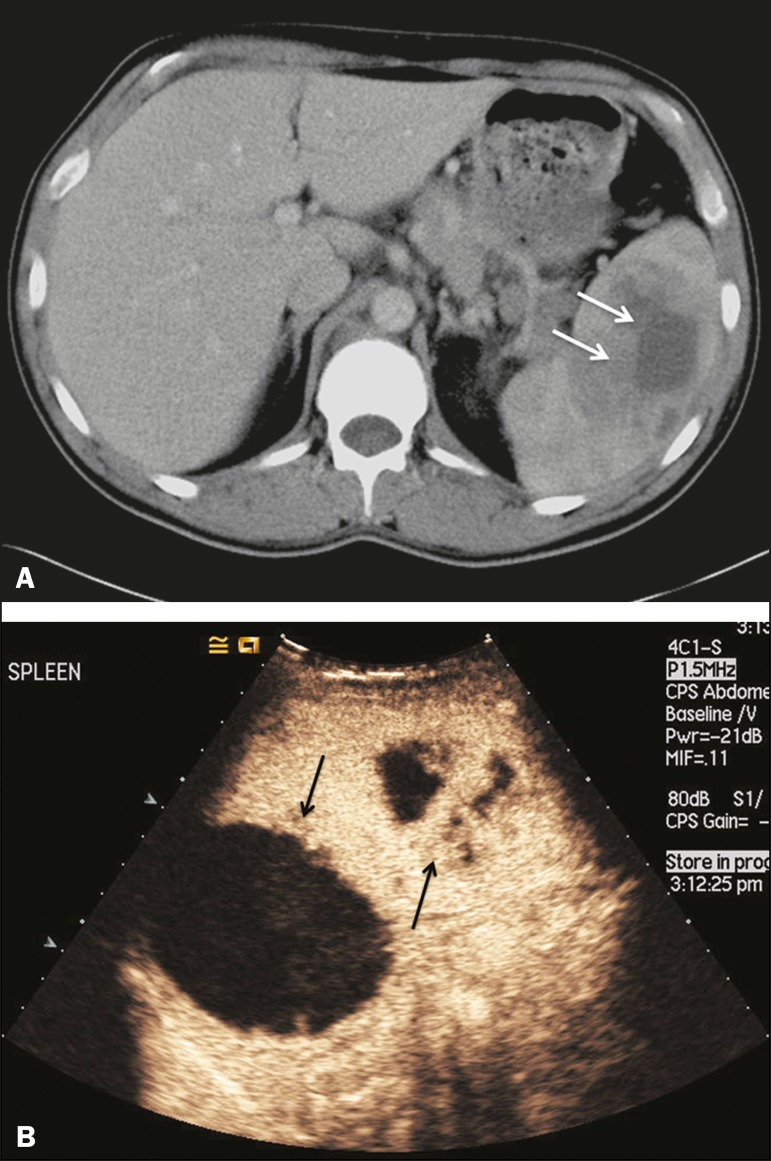
Abscess. **A:** Contrast-enhanced CT scan showing a hypodense splenic lesion with a thick, irregular wall (arrows). **B:** CEUS showing no internal enhancement and an irregular wall in the larger abscess, with thickened, enhancing septations in the smaller abscess (arrows).

At least 70% of splenic abscesses result from the hematogenous spread of infection from elsewhere ^(8)^. The most common primary sources of such infection are the following: the endocardium (endocarditis); the urinary tract; a surgical or traumatic wound; and the appendix (appendicitis). Splenic abscesses are typically cystic, with a thick, irregular wall, and contain debris and septations ^(9)^. CEUS appearances can vary depending on the number of internal septations. Generally, there is hyperenhancement of the wall and enhancement of the internal septations (Figure 3). Again, as with a cyst, there should be no enhancement of the debris or fluid/necrotic areas, the wall irregularity and enhancement distinguishing an abscess from a cyst.

### Granulomatous disease of the spleen

**Figure 4:**
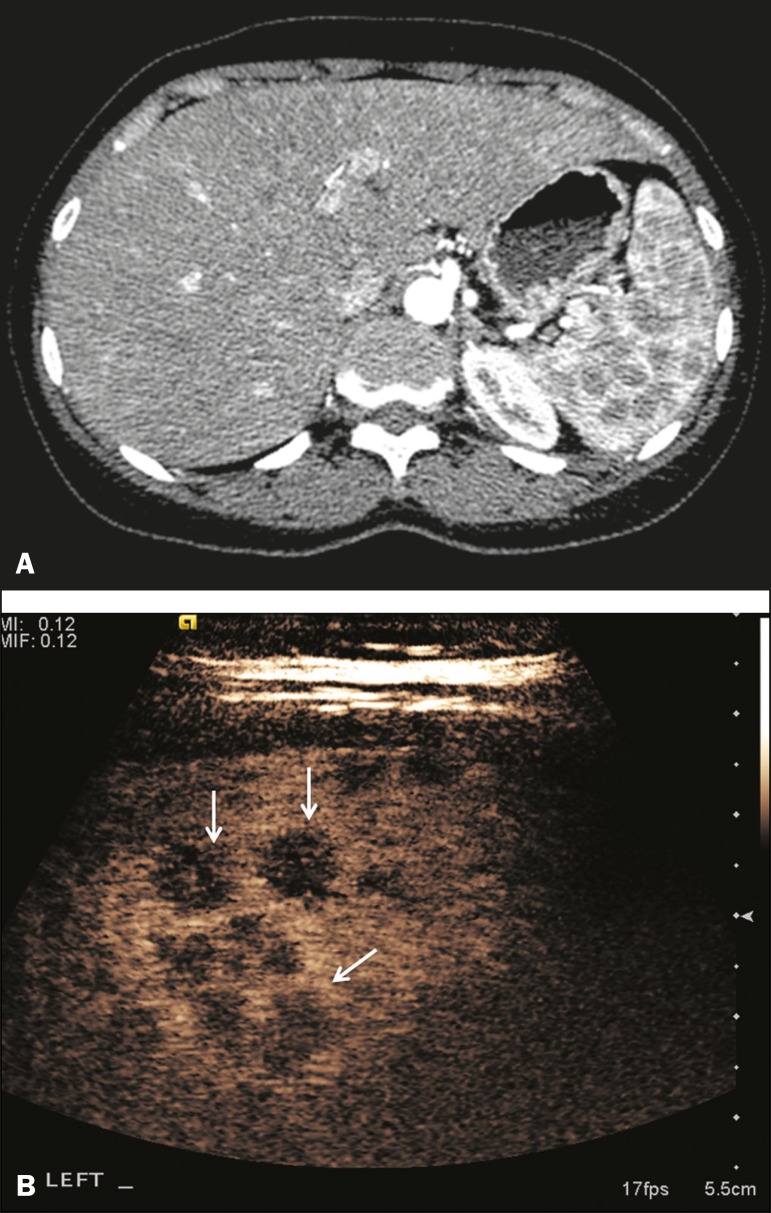
Granulomatous disease. **A:** Contrast-enhanced CT scan showing multiple low-density lesions within the spleen, less obvious in the liver. **B:** Following microbubble contrast administration, the splenic lesions remain hypovascular throughout the CEUS examination but do show some internal vascular enhancement (arrows). The patient depicted had a history of biopsy-proven sarcoidosis

Granulomatous diseases such as sarcoidosis and tuberculosis can involve the spleen; for example, 60% of sarcoidosis patients have splenic involvement ^(10)^. Usually, this will manifest as splenomegaly with multiple nodules and evidence of sarcoidosis elsewhere ^(10)^, as depicted in Figure 4.

### Splenic infarction

**Figure 5:**
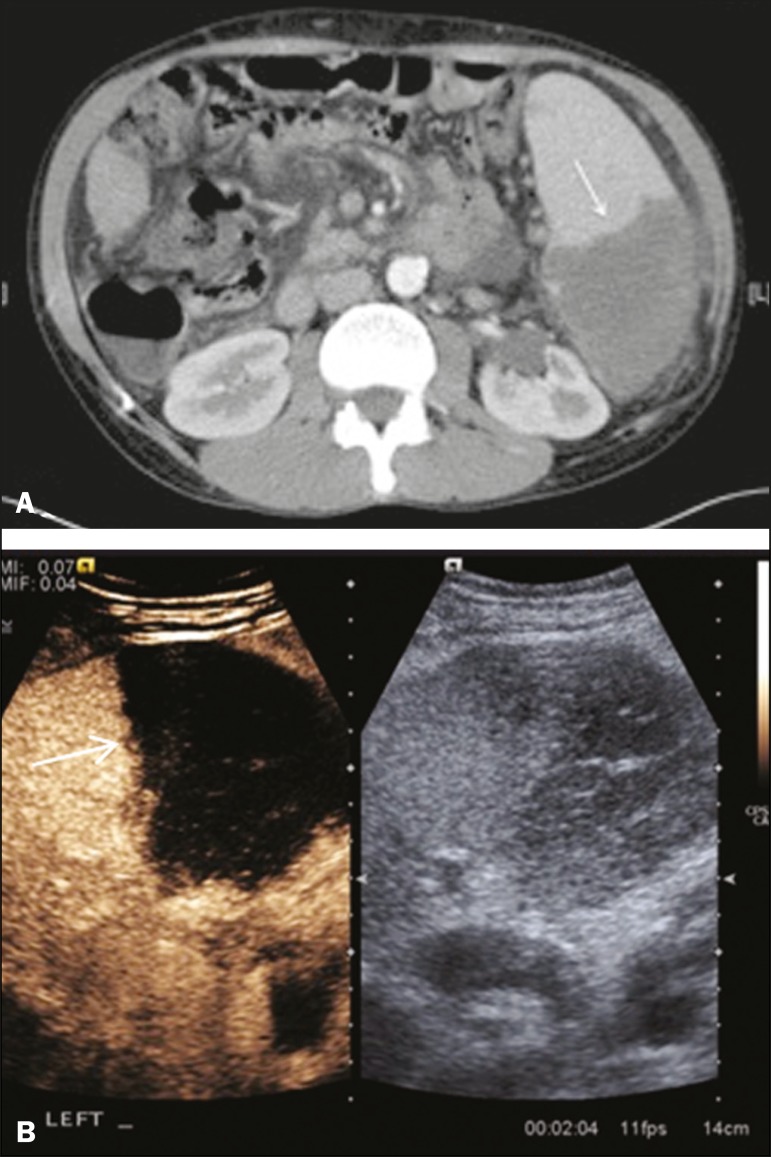
Splenic infarction. **A:** Contrast-enhanced CT scan, in a pre-assessment for liver transplantation, showing splenomegaly with sharply demarcated low-density area in the posterior-inferior pole of the spleen (arrow). **B:** A split-screen view. Conventional ultrasound (right side of the screen) shows a hypoechoic, heterogeneous lower pole of the spleen, and CEUS (left side of the screen) shows a total absence of enhancement of the lower pole of the spleen, confirming a large wedge-shaped infarction (arrow). The patient was found to have aortic valve endocarditis.

Splenic infarction can arise as a result of a number of etiologies. The vast majority are either infiltrative hematologic diseases that cause congestion of the splenic circulation by abnormal cells or thromboembolic conditions that produce obstruction of the large vessels ^(11)^. The areas of infarction are usually well marginated and wedge-shaped, and there is often evidence of an underlying cause. On CT, an infarction might not be distinguishable from an abscess or an infiltrative process, the subtle gray-scale ultrasound changes not clearly establishing the cause. On CEUS, the distinction between the wedge-shaped, well-marginated appearance of an infarction and the irregular borders of an abscess is easily made (Figure 5).

### Splenic trauma

**Figure 6:**
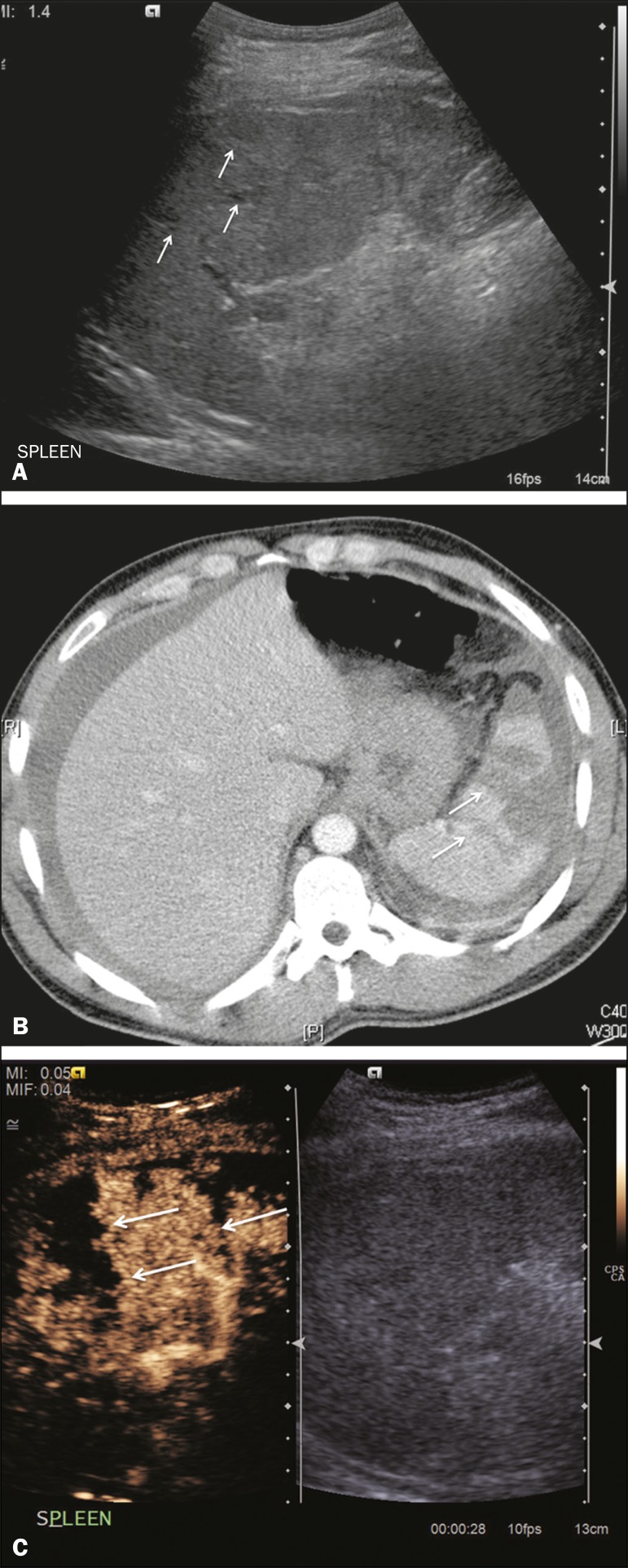
Splenic trauma. **A:** B-mode ultrasound showing subtle heterogeneous abnormalities within the spleen (arrows). **B:** Contrast-enhanced CT scan showing irregular areas of hypodensity within the spleen in keeping with intraparenchymal fractures (arrows). **C**: A split-screen view. Following microbubble contrast administration, CEUS showed areas of heterogeneous contrast uptake (fracture plane/hematoma) with curvilinear and wedge-shaped areas of hypovascularity extending to the hilum, consistent with fractures (arrows).

It is challenging to diagnose traumatic splenic injury solely on the basis of B-mode ultrasound images, and contrast-enhanced CT is often the modality of choice for the assessment of the spleen after blunt abdominal trauma. However, it must be noted that the addition of microbubble contrast to the ultrasound examination makes the images equivalent to those of contrast-enhanced CT ^(12)^. CEUS can be used at the bedside to delineate splenic injury in a trauma patient and is useful in the follow-up imaging evaluation of such patients, to avoid the ionizing radiation exposure of repeated CT examinations (Figure 6).

### Splenic lymphoma

**Figure 7:**
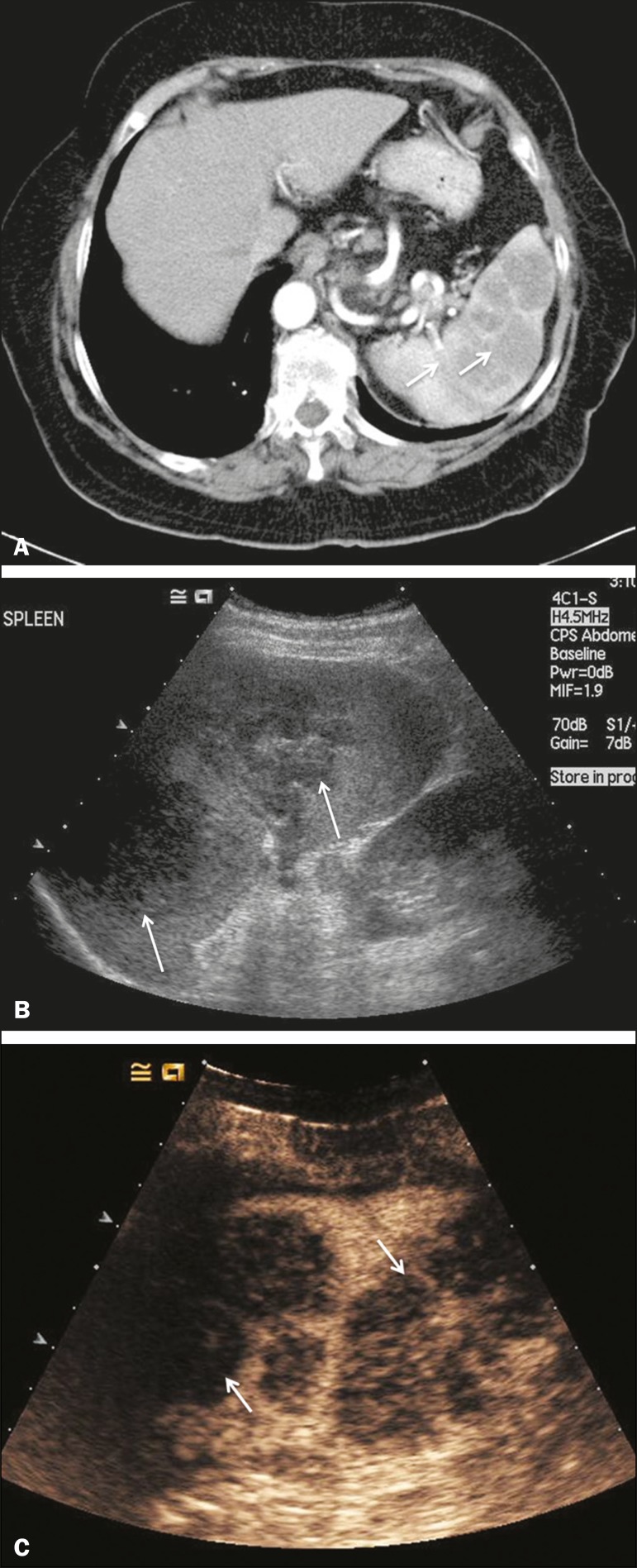
Multiple splenic lymphoma. **A:** Contrast-enhanced CT scan showing hypodense, lobulated soft-tissue lesions (arrows) in a patient who presented with abdominal pain and distension. **B:** B-mode ultrasound showing that lesions (arrows) are ill-defined and of mixed reflectivity. **C:** Following microbubble contrast administration, CEUS showed early arterial enhancement and rapid washout (arrows) in the venous phase. The appearances are suggestive of either metastatic disease or lymphoma. Following splenectomy, this was proven to be a case of lymphoma.

**Figure 8:**
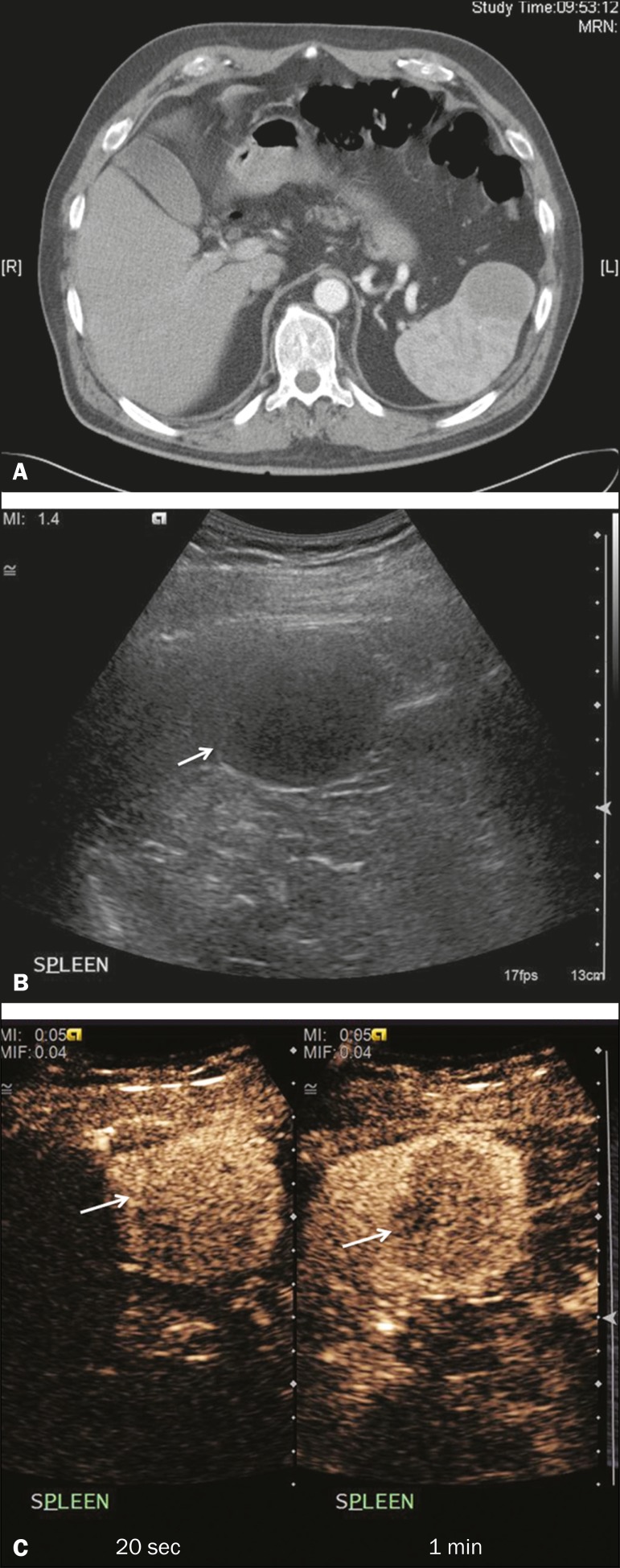
Splenic fibrosis. **A:** In the routine follow-up CT of a lymphoma patient, a new, hypodense lesion was observed. **B:** B-mode ultrasound showing a hypoechoic lesion (arrow). **C:** CEUS showing increased arterial enhancement at 20 s and washout after 1 min (arrows). The suggested diagnosis was disease infiltration, and splenectomy was performed. The lesion was histologically proven to be focal fibrosis on a background of chronic inflammatory changes.

**Figure 9:**
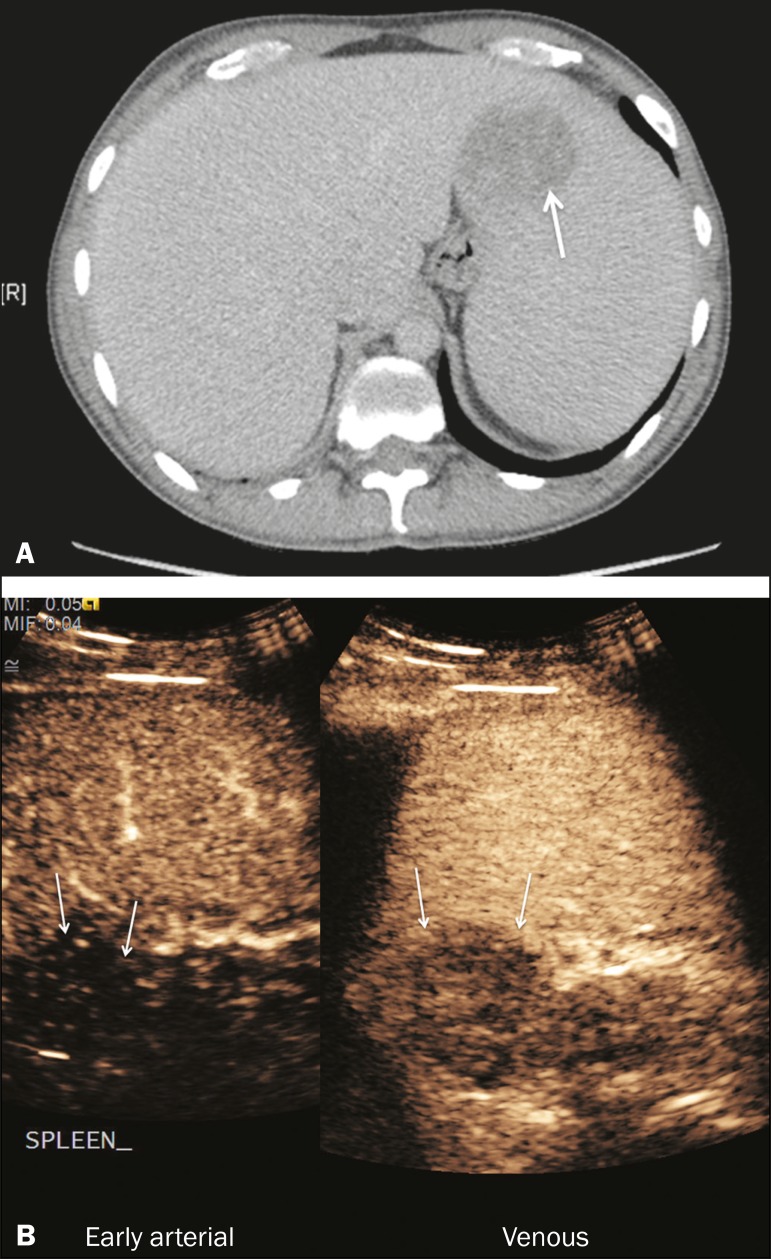
Hairy-cell leukemia. **A:** CT scan showing a rounded, hypodense soft-tissue lesion (arrow). **B:** On CEUS, the lesion shows poor uptake in the arterial phase (arrows) and is hypodense in comparison with the surrounding tissue, although increasing in contrast in the venous phase (arrows).

Lymphoma is the most common splenic malignancy, and splenic lymphoma is usually the initial manifestation of systemic lymphoma, 10% to 30% of patients having splenic involvement at presentation ^(13)^. The imaging appearances range from diffuse involvement to miliary nodules, mu tiple focal lesions and a solitary mass (Figure 7). Larger lesions can appear cystic due to central necrosis. The vascular pattern of lymphoma is thought to be distinct from that of other focal malignant lesions of the spleen, with preservation of less disordered vessels, particularly in the late phase of enhancement. However not all splenic lesions in lymphoma patients show disease infiltration, and radiologists should be aware of other entities that can resemble focal splenic infiltration, such as focal fibrosis (Figure 8). A distinct form of low-grade non-Hodgkin lymphoma is hairy-cell leukemia (Figure 9), which accounts for approximately 2% of all cases of leukemia ^(14)^.

### Splenic metastases

**Figure 10:**
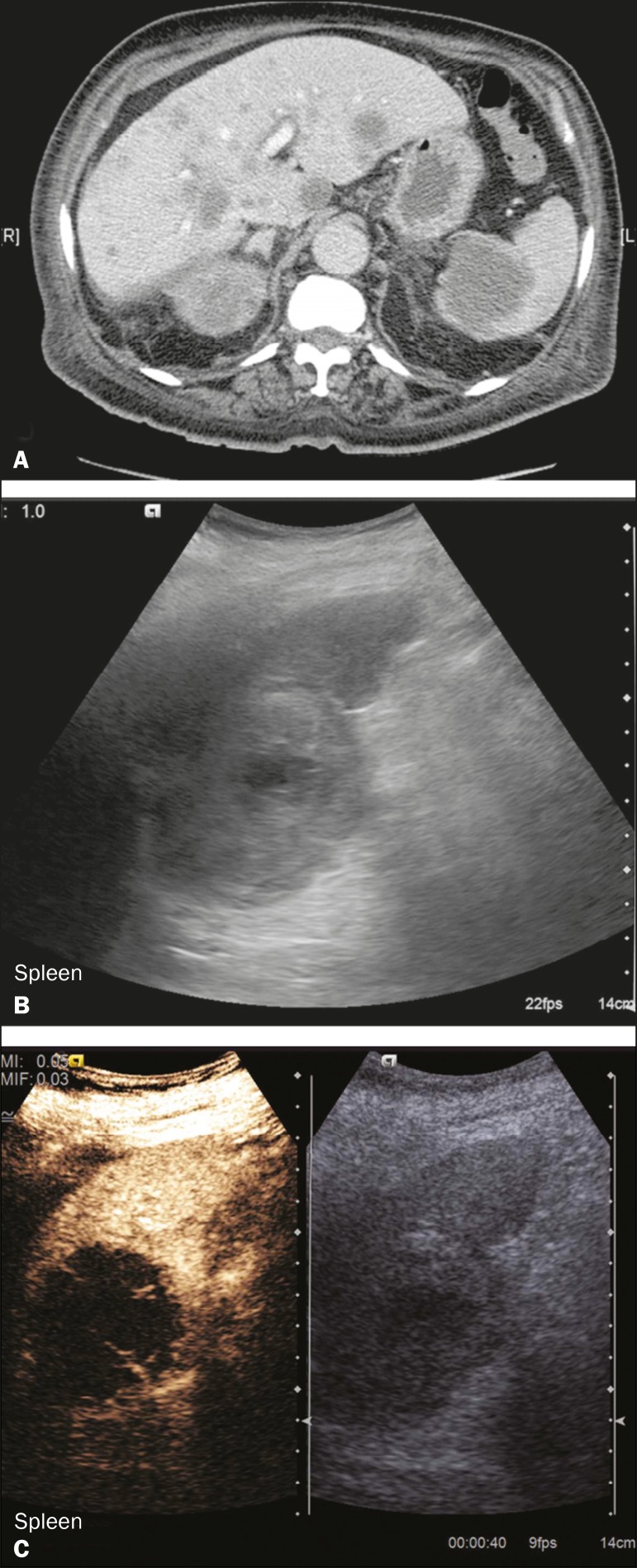
Splenic metastasis. **A:** CT scan showing several hypodense lesions in the liver and spleen, a large dominant lesion, with central necrosis, being evident in the spleen. **B:** B-mode ultrasound showing that the dominant lesion (within the spleen) was ill-defined and heterogeneous, with possible central necrosis. **C:** A split-screen view. CEUS showed that the lesion was largely avascular and necrotic, with some septations. This appearance is highly suggestive of necrotic metastases. This patient had disseminated metastatic disease, the primary tumor being identified as renal cell carcinoma.

Splenic metastases are rare and usually asymptomatic. The most common primary neoplasms giving rise to such metastases are breast cancer, lung cancer, colorectal cancer, ovarian cancer, and malignant melanoma ^(15)^. On imaging, splenic metastases typically appear as multiple solid nodules, occasionally appearing as cystic nodules, particularly if the primary site is the ovary. On CEUS, the behavior of splenic metastases is similar to that of hepatic metastases, featuring washout and chaotic vessels (Figure 10).

### Splenic hemangioma

**Figure 11:**
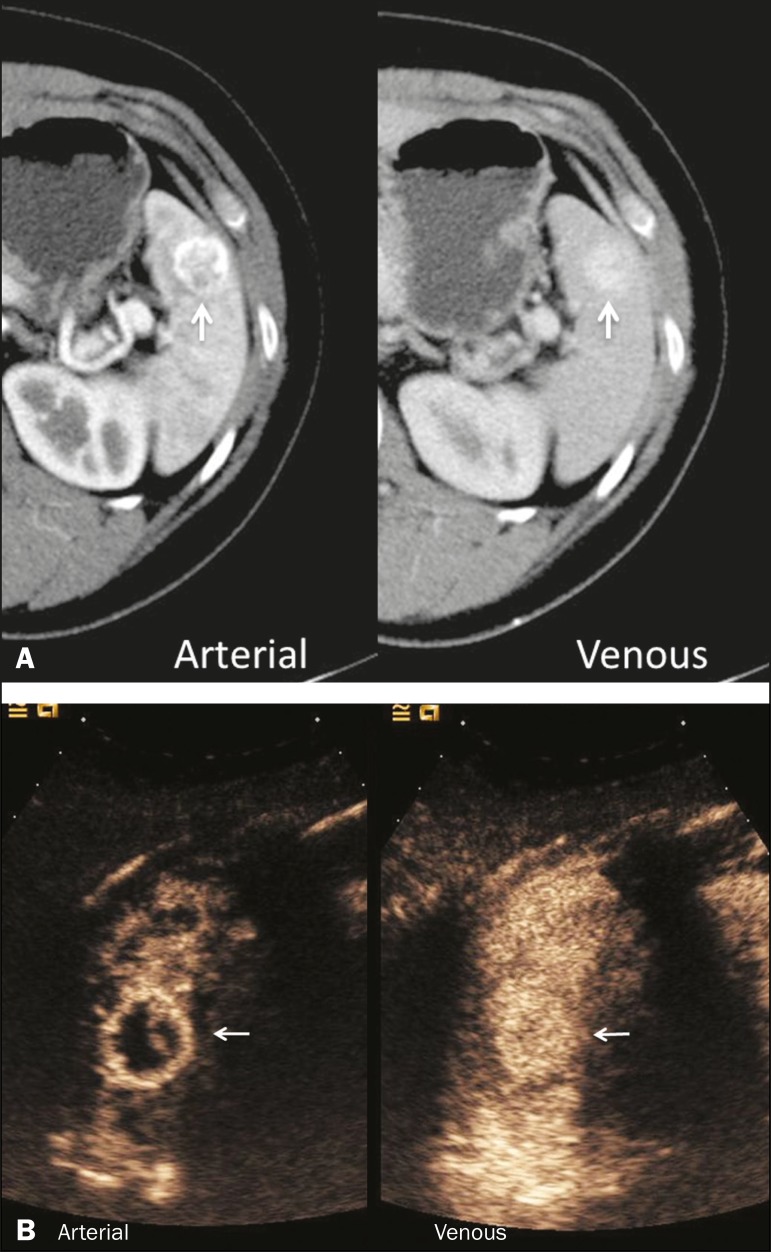
Splenic hemangioma. **A:** Contrast-enhanced CT scan showing peripheral enhancement and delayed filling of the splenic lesion, characteristic of a splenic hemangioma (arrows). **B:** CEUS showing a classic splenic hemangioma (arrows) with avid peripheral enhancement in the early phase (23 s) with some internal filling, similar to the arterial-phase filling seen on CT. After approximately 40 s, the hemangioma filled completely, with an enhancement pattern similar to that of the surrounding spleen. Note the similarity to the venous-phase CT scan of the same patient.

Splenic hemangioma is congenital and is the most common benign lesion of the spleen. It is typically an incidental finding, most often occurring in adults between 30 and 50 years of age ^(16)^. The prevalence of splenic hemangioma at autopsy ranges from 0.3% to 14%. When large, a hemangioma can evolve to thrombosis, infarction, or rupture ^(17)^. Hemangioma can also be multiple, as in Klippel-Trenaunay-Weber syndrome. Capillary hemangiomas are usually small and echogenic on B-mode ultrasound, whereas they show isoechoic enhancement on CEUS. Large cavernous hemangiomas can have a combination of solid and cystic parts, with partial or complete centripetal filling (Figure 11).

### Splenic hamartoma

**Figure 12:**
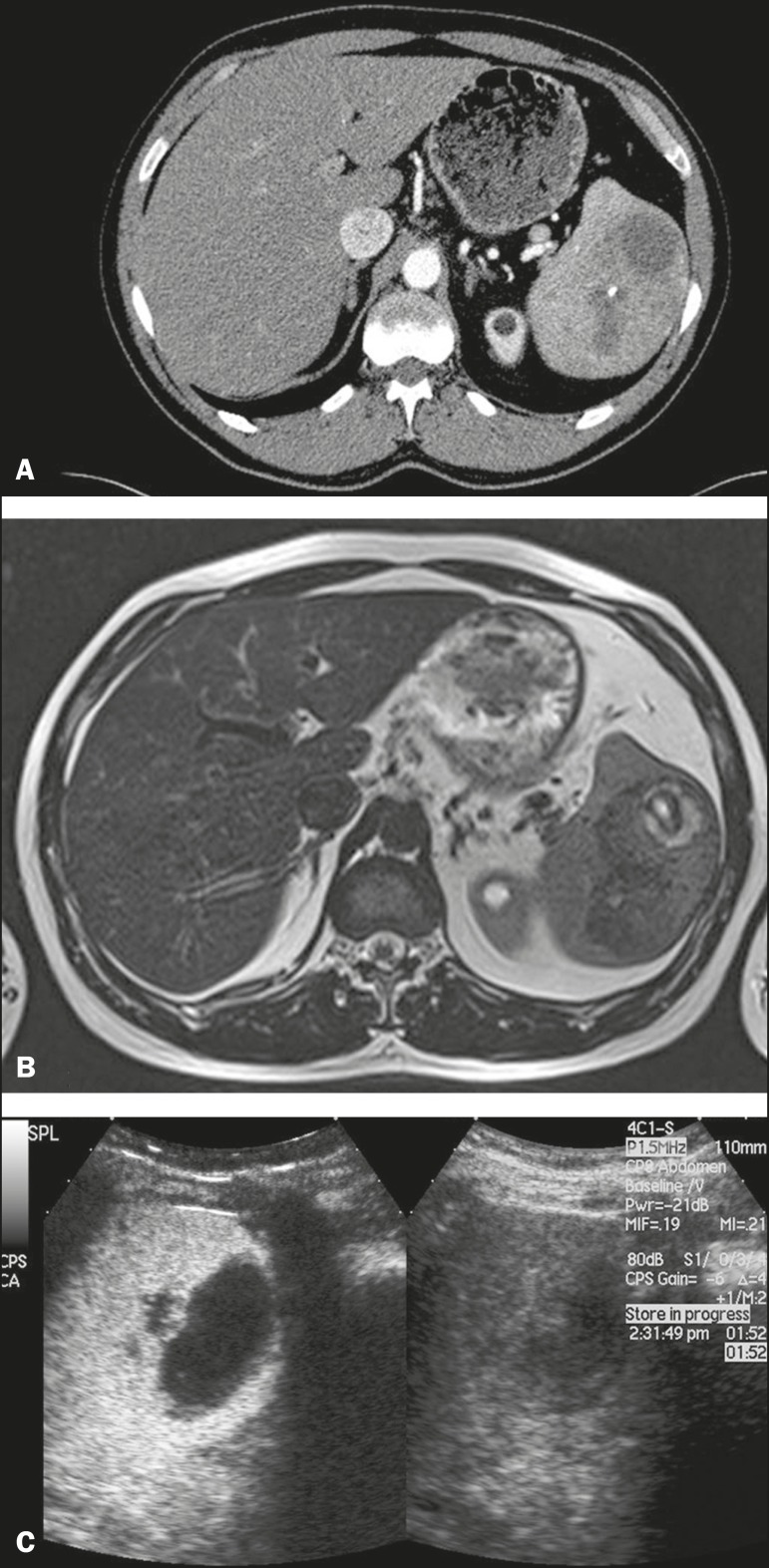
Splenic hamartoma. **A:** Venous-phase contrast-enhanced CT scan showing a complex enhancing mass with central areas of fluid density and a focus of calcification, causing alteration of the contour of the spleen and retraction of the capsule. **B:** T2-weighted MRI scan showing that the lesion is heterogeneous, with an isointense to hyperintense signal. **C:** A split-screen view. CEUS revealed that the lesion showed no internal enhancement, consistent with a splenic hamartoma, as was all of the imaging.

Splenic hamartoma, also known as splenoma, splenic adenoma, or nodular hyperplasia, is a rare benign lesion that can occur at any age ^(16)^ and can be associated with hamartoma elsewhere in the body, particularly in conjunction with tuberous sclerosis. Although splenic hamartoma is likely a focal developmental disturbance, it has been suggested that it can arise from a proliferative process or as a traumatic lesion. A splenic hamartoma is typically a well-defined, solid, nodular lesion that compresses the surrounding splenic tissue. In some cases, it is cystic or contains calcifications. When a splenic hamartoma is solid, CEUS shows a varying degree of enhancement in the late phase; when cystic, it presents like any other cystic structure and shows no internal enhancement (Figure 12).

### Splenic lymphangioma

**Figure 13:**
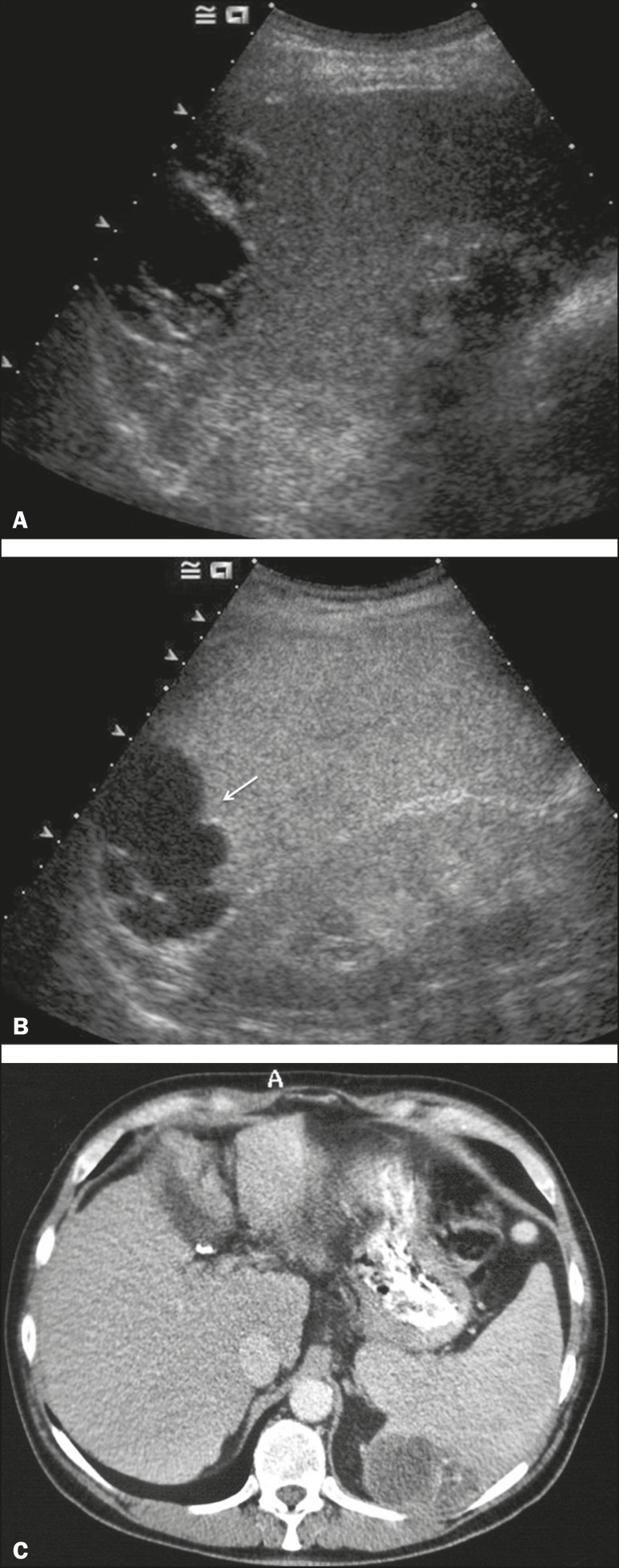
Splenic lymphangioma. **A:** B-mode ultrasound showing a lobulated well-defined abnormality in the upper aspect of the spleen, with septations and calcifications. **B:** On CEUS, the lesion is more conspicuous, the lobulated outline and septations being clearly demonstrated (arrow). The central aspect of the lesion shows no contrast enhancement. **C:** The corresponding CT scan confirmed the septations and foci of calcification.

Splenic lymphangioma is a rare benign tumor of unknown origin that is related to, although much less common than, a hemangioma and is seen predominantly in children. A splenic lymphangioma is normally asymptomatic. However, when the tumor is large, it can be complicated by bleeding, consumptive coagulopathy, or portal hypertension. Splenic lymphangiomas are usually subcapsular and typically appear on ultrasound as multiple cysts, often with internal debris or septations ^(16)^ Figure 13). On CEUS, the septations and capsule can show enhancement. On CT, splenic lymphangiomas appear as thin-walled, low-attenuation masses without contrast enhancement. Mural calcifications are occasionally present.

### Splenic artery pseudoaneurysm

**Figure 14:**
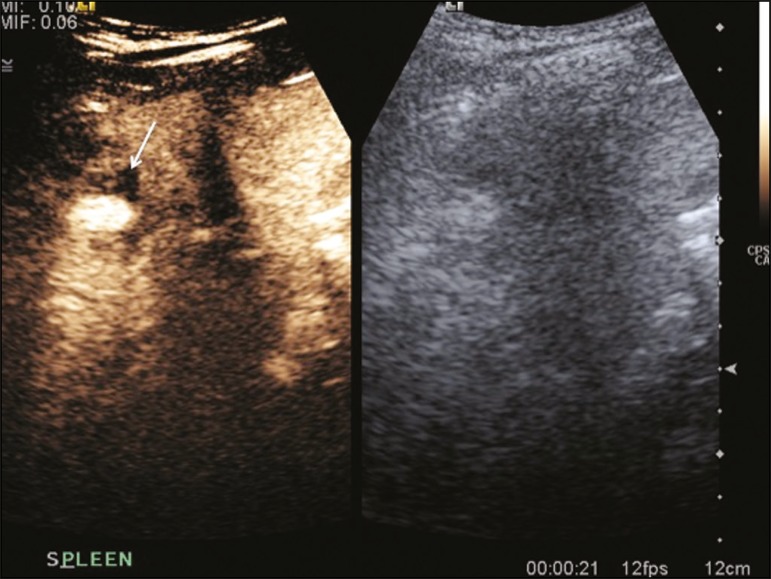
Splenic pseudoaneurysm identified during the follow-up evaluation of a patient who had experienced low-energy blunt abdominal trauma, with no evidence of pseudoaneurysm formation in the initial CT scan. A split-screen view. CEUS showed a focus of hyperenhancement within the fracture plane, consistent with a pseudoaneurysm.

Splenic artery pseudoaneurysm is a rare entity that usually results from trauma, pancreatitis, or surgery. If left untreated, a splenic pseudoaneurysm has a risk of rupture of up to 37% and, if ruptured, a mortality rate of up to 90%; when detected, the appropriate treatment can be endovascular or surgical, depending on the size of the pseudoaneurysm, although its size is not predictive of the risk of hemorrhage ^(18,19)^. CEUS plays a significant role in the imaging investigation of patients suspected of developing a pseudoaneurysm (1 day to 4 months after a traumatic event). The CEUS imaging findings are those of a rounded well-defined lesion usually lying within the fracture plane (when post-traumatic) and demonstrate vascular enhancement similar to that seen on contrast-enhanced CT, CEUS having the advantage of real-time dynamic imaging ^(20)^, as illustrated in Figure 14.

### Splenunculi

**Figure 15:**
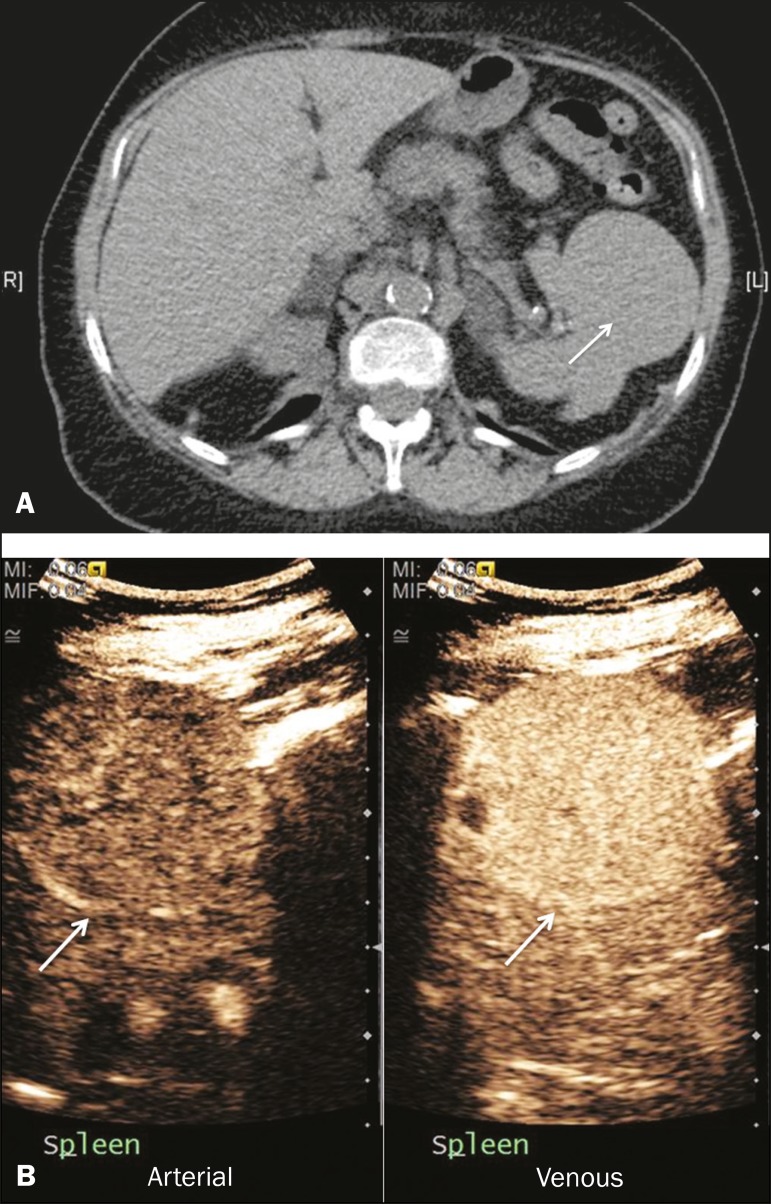
Splenunculi. **A:** Non-contrast-enhanced CT scan, performed for hematuria workup, showing a large, well-defined lesion adjacent the spleen (arrow). **B:** A split-screen view. CEUS showed that the lesion (arrows) was enhanced in a manner identical to that of the adjacent splenic parenchyma, confirming that this was a large splenunculus.

Splenunculi are variants of normality, identified in 30% of autopsies ^(7)^. On imaging, splenunculi typically present adjacent to the splenic hilum; they can be single or multiple and are usually ≤ 2 cm in diameter. Splenunculi can be ectopic and can occur in a variety of locations, including the pancreas and scrotal sac. On CT and ultrasound, splenunculi appear as well-defined nodules, with density similar to that of the spleen itself (Figure 15). On B-mode ultrasound, they have the same echotexture and echogenicity as the spleen. On color Doppler ultrasound, an arterial hilum can be seen. The principal differential diagnoses are a tumor in the tail of the pancreas and an abnormal lymph node. On CEUS, splenunculi present enhancement features similar to those of the rest of the splenic parenchyma. The enhancement pattern is an important characteristic to differentiate a splenunculus from a pancreatic tail tumor or an abnormal lymph node ^(5)^.

## CONCLUSION

CEUS is a powerful, accessible tool for the study of the spleen. Comparing CEUS with B-mode ultrasound, the addition of microbubble contrast increases the conspicuity of the majority of incidentally identified splenic lesions and can be used with confidence to determine the nature of cysts, hemangiomas, infarctions, and abscesses, as well as to facilitate the differentiation between benign and malignant lesions. CEUS improves trauma imaging of the spleen, making it a practical tool for increasing diagnostic reliability. The estimation of the extent of traumatic lesions with CEUS is far more accurate than that achieved with B-mode ultrasound and similar to that achieved with contrast-enhanced CT. CEUS of the spleen can also be used in follow-up evaluations to identify complications associated with traumatic laceration of the spleen, including pseudoaneurysm formation, and allows ionizing radiation-free assessment of resolution of the injury.
